# Identification and evaluation of *Lonicera japonica* flos introduced to the Hailuogou area based on ITS sequences and active compounds

**DOI:** 10.7717/peerj.7636

**Published:** 2019-09-03

**Authors:** Haiyan He, Dan Zhang, Jianing Gao, Theis Raaschou Andersen, Zishen Mou

**Affiliations:** 1 Key Laboratory of Mountain Surface Processes and Ecological Regulation, Institute of Mountain Hazards and Environment, Chinese Academy of Sciences, Chengdu, People’s Republic of China; 2 University of Chinese Academy of Sciences, Beijing, People’s Republic of China; 3 Centre for Applied Research & Development, VIA University College, Horsens, Denmark; 4 State Environmental Protection Key Laboratory of Synergetic Control and Joint Remediation for Soil & Water Pollution (Chengdu University of Technology), Chengdu, People’s Republic of China; 5 State Key Laboratory of Geohazard Prevention and Geoenvironment Protection, Chengdu, People’s Republic of China

**Keywords:** *Lonicera japonica* Thumb., ITS sequence, Identification, Chlorogenic acid, Luteoloside, Evaluation

## Abstract

*Lonicera japonica* flos (LJF), the dried flower buds of *L. japonica* Thunb., have been used in traditional Chinese herbal medicine for thousands of years. Recent studies have reported that LJF has many medicinal properties because of its antioxidative, hypoglycemic, hypolipidemic, anti-allergic, anti-inflammatory, and antibacterial effects. LJF is widely used in China in foods and healthcare products, and is contained in more than 30% of current traditional Chinese medicine prescriptions. Because of this, many Chinese villages cultivate LJF instead of traditional crops due to its high commercial value in the herbal medicine market. Since 2005, the flower buds of *L. japonica* are the only original LJF parts considered according to the Chinese Pharmacopoeia of the People’s Republic of China. However, for historical and commercial reasons, some closely related species of *Lonicera Linn*. continue to be mislabeled and used as LJF. Currently, there are hundreds of commercial varieties of LJF on the market and it is difficult to choose fine LJF varieties to cultivate. In this study, a total of 21 varieties labeled as LJF on the market were planted in the Hailuogou area. In order to choose the optimum variety, internal transcribed spacer (ITS) sequence alignment analysis was used to test whether the 21 varieties were genuine LJF or not. Cluster analysis of active components based on the content of chlorogenic acid and luteoloside in flower buds, stems and leaves was used to evaluate the quality of the varieties. Results demonstrated that four of the varieties were *L. macranthoides* Hand.-Mazz., while the other 17 varieties were *L. japonica*, and genuine LJF. The ITS sequence analysis was proven to be highly effective in identifying LJF and *Lonicerae* flos. Among the 17 *L. japonica* varieties, the amounts of chlorogenic acid and luteoloside in flower buds, stems and leaves were significantly different. Based on the cluster analysis method, the variety H11 was observed to have the highest level of active components, and is therefore recommended for large-scale planting in the Hailuogou area.

## Introduction

*Lonicera japonica* flos (LJF, honeysuckle), the dried flower buds of *L. japonica* Thunb. (Caprifoliaceae), have been used in traditional Chinese medicine for thousands of years. Recent studies have found that LJF has many medicinal properties (Shang et al., 2011) due to its antioxidant (Hsu et al., 2016; Seo et al., 2012; Dung et al., 2011), hypoglycemic and hypolipidemicc (Wang, Zhao & Liu, 2017), anti-allergic (Tian et al., 2012; Oku et al., 2011), anti-inflammatory (Hsu et al., 2016; Lee et al., 2011; Ryu et al., 2010) and antibacterial effects (Xiong et al., 2013; Han et al., 2014). Prior to 2005, *L. japonica* and three closely related species (*L. hypoglauca* Miq., *L. confunsa* DC. and *L. dasystyla* Rhed.) were described as the original LJF plants in the Pharmacopoeia of the People’s Republic of China (Ch.P) (Ministry of Health of the People’s Republic of China, Commission of Pharmacopoeia, 1977, 1985, 1990, 1995; Chinese Pharmacopoeia Commission, 2000). Since 2005, only *L. japonica* has been considered in the Ch.P (Chinese Pharmacopoeia Commission, 2005, 2010, 2015). However, the dried flower buds of *L. hypoglauca*, *L. confunsa*, *L. fulvotomentosa* Hsu et S.C. Cheng, *L. hypoglauca* Miq. ssp. Nudiflora Hsu et H.J. Wang, *L. nubium* Hand.-Mazz., *L. macranthoides* Hand.-Mazz, *L. similis* Hemsl. (Wang et al., 2014; Hu et al., 2012; Qian et al., 2007; Li et al., 2006) continue to be mislabeled as LJF in China, but are not well distinguished in practical applications.

In recent years, the demand of LJF in the fields of pharmacy (Shang et al., 2011), cosmetics (Ning et al., 2012), and health food (Feng et al., 2018; Li et al., 2014; Wang, Clifford & Sharp, 2008) has increased. Over 30% of current traditional Chinese medicine prescriptions contain LJF, and the commercial value of LJF in herbal medicine markets has increased over 400% in recent years (Yuan et al., 2012). The economic value of cultivating LJF is much higher than that of traditional crops, and many Chinese villages have started to cultivate LJF instead of traditional crops to increase income. However, commercial varieties can be named independently by the cultivator because of a lack of supervision, resulting in hundreds of commercial varieties of LJF on the market. Thus, it is challenging to choose fine LJF varieties to plant. For cultivars especially, the problem still exists of distinguishing landraces and wild germplasm morphologically from each other.

In the past four decades, several techniques based on molecular markers have been developed to identify and evaluate plant germplasm. Restriction fragment conformation polymorphism (RFLP), randomly amplified polymorphic DNA (RAPD), simple sequence repeat (SSR) and amplified fragment length polymorphism (AFLP) are widely used. Among these methods, RFLP is a co-dominant marker and can detect high polymorphism (Outram et al., 2014), but it has a strict requirement for DNA purity. The bands obtained are complex and difficult to interpret (Ren et al., 2017). RAPD is a dominant genetic marker but cannot distinguish heterozygotes from homozygotes, and has poor reproducibility and low reliability (Nagaoka & Ogihara, 1997). SSR based markers are predominantly co-dominant, but they may have high development costs and can sometimes generate false-positive (Peakall et al., 1998) or false negative results (Devos et al., 1995). AFLP markers have strong polymorphism, are stable and have reproducible results (Dorji & Yapwattanaphun, 2015). However, ALFP is expensive and requires isotopic testing and high purity of DNA (Ren et al., 2017). Compared with the above-mentioned methods, internal transcribed spacer (ITS) sequence alignment analysis of nrDNA is an ideal molecular marker technique. ITS is highly repetitive with a large number of copies and the length is generally less than 700 bp (including 5.8S rDNA), which is beneficial for amplification, cloning and sequencing (Baldwin et al., 1995; Elder & Turner, 1995). The 18S, 5.8S, 26S rDNA genes are highly conserved and can be amplified and sequenced using universal primers complementary to their sequences (Ghada et al., 2009). The nucleotide sequence changes dramatically, which provides rich phylogenetic information (Schmidt & Schilling, 2000). In addition, ITS analysis is simple and cost-effective. It is widely used in identification and phylogenetic analysis of original plants of Chinese medicinal herbs, such as *Acori Tatarinowii Rhizoma* (dried rhizome of *Acorus tatarinowii* Schott) (Lam et al., 2016), *Corni Fructus* (ripe fruit of *Cornus officinalis* Siebold et Zucc.) (Hou et al., 2013), *Fallopia multiflora* (dried rhizome of *Fallopia multiflora* (Thunb.) Haraldson.) (Zheng et al., 2009), Pulsatillae radix (dried root of *Pulsatilla chinensis* (Bge.) Regel) (Shi et al., 2017), and saffron (dried stigmas of *Crocus sativus* L.) (Huang et al., 2015). The nrDNA ITS sequences of *L. japonica* differed significantly from that of related species (Peng et al., 2010; Hu et al., 2012; Wang et al., 2014).

Currently, there are few studies on the identification of LJF commercial varieties, especially in agricultural introduction and planting. In this study, 21 varieties called LJF with a certain market share were introduced to farmers in the Hailuogou area to plant. The yield and quality of the 21 varieties were significantly different. ITS sequence alignment analysis was used to identify the authenticity of the LJF varieties. High-performance liquid chromatography (HPLC) was applied to detect the content of active components (chlorogenic acid and luteoloside) in flower buds, stems and leaves. The Cluster analysis method based on active components was used to evaluate the medicinal value of introduced varieties and in selecting suitable varieties for later large-scale planting.

## Materials and Methods

### Study area

The study area is located in Baozi village, Hailuogou area, Tibetan Autonomous Prefecture of Ganzi, Sichuan Province, P. R. China (Longitude of 102.11E and Latitude of 29.63N). The area is located in the western Qinghai Tibet Plateau climate transition zone and is characterized by a subtropical warm and humid monsoon climate. The annual mean temperature is 12.7 °C and annual mean precipitation is 1,050.3 mm (Wu et al., 2013). The relative humidity is 66% and yearly sunlight hours are 1,161.9 h.

### Sampling, DNA extraction, PCR amplification and sequencing

Two-year-old seedlings of 21 varieties (H1–H21) were planted in the study area in September 2014. Samples of young leaves were harvested in October 2015 (Table 1). From each variety, three plant samples that shared the same morphology were collected. The total genomic DNA was extracted from fresh leaves using a modified CTAB method (Uddin et al., 2014). PCR amplification of DNA was carried out in a 25 μL volume for all samples, and included 19 μL 1.1× PCR Mix, four μL DNA (60 ng/μL) and two μL 10 μmol/L mixed primers with the equal volume mixing universal primers ITS1 (5′-AGAAGTCGTAACAAGGTTTCCGTAGG-3′) and ITS4 (5′-TCCTCCGC TTA TTGATATGC-3′) (Wang et al., 2014). The ITS sequence was amplified at 98 °C for 2 min followed by five touchdown cycles of 98.0 °C for 10 s, 58.4 °C for 10 s (every cycle lowered 1 °C), 72.0 °C for 10 s; and then followed by 25 cycles of 98.0 °C for 10 s, 53.4 °C for 10 s, 72.0 °C for 10 s, and a final extension at 72 °C for 10 min. All PCR products were detected by electrophoresis on 2% agarose gel in 0.5× TAE buffer, stained with ethidium bromide and imaged using a Gel Imaging System. The PCR amplification products were found to be highly specific and could be sequenced directly. Forward and reverse sequencing was completed by Shanghai Sangon Biotech Corporation. PCR amplification and sequencing were repeated in triplicate.

**Table 1:** GenBank number and sources of test samples and reference sequences in GenBank.

No.	Name	Sources of introduction	GenBank No.	Reference sequence in GenBank
H1	Zhongjin 1#	Fei County, Shandong Province	MG491249	KX394609.1, JQ780991.1, JQ731715.1
H2	Red bud	Fei County, Shandong Province	MG491250	KF160906.1, JQ780986.1, KX394609.1
H3	Mengjin 1#	Pingy County, Shandong Province	MG491251	KX394609.1, JQ780991.1, JQ731712.1
H4	Mengjin 2#	Pingy County, Shandong Province	MG491252	KF160906.1, JQ780986.1, KX394609.1
H5	Mengjin 3#	Pingy County, Shandong Province	MG491253	KX394609.1, JQ780991.1, JQ731715.1
H6	Jizhua flower	Pingy County, Shandong Province	MG491254	KX394609.1, JQ780991.1, JQ731715.1
H7	Jiufeng 1#	Pingy County, Shandong Province	MG491255	JQ780986.1, KX394609.1, KF160906.1
H8	Tefeng 1#	Cangshan County, Shandong Province	MG491256	KX394609.1, JQ780991.1, JQ731715.1
H9	Tefeng 2#	Cangshan County, Shandong Province	MG491257	KX394609.1, JQ780991.1, JQ731715.1
H10	Lufengwang 1#	Linyi City, Shandong Province	MG491266	KF160887.1, JQ731716.1, FJ372922.1
H11	Lufengwang 3#	Linyi City, Shandong Province	MG491258	KX394609.1, JQ780991.1, JQ731715.1
H12	Lufengwang 5#	Linyi City, Shandong Province	MG491259	JQ780986.1, KX394609.1, KF160906.1
H13	Lufengwang 6#	Linyi City, Shandong Province	MG491260	KF160912.1, JQ780990.1, KX394609.1
H14	Jufeng	Linyi City, Shandong Province	MG491261	KX394609.1, JQ780991.1, JQ731715.1
H15	Jinfeng 1#	Fengqiu County, Henan Province	MG491262	KX394609.1, JQ780991.1, JQ731715.1
H16	Shipan Flower	Jianyang City, Sichuan Province	MG491263	KX394609.1, JQ780991.1, JQ731715.1
H17	Muchuan 1#	Muchuan County, Sichuan Province	MG491267	FJ372919.1, KF160915.1, KF160887.1
H18	Muchuan 2#	Muchuan County, Sichuan Province	MG491268	FJ372919.1, KF160915.1, KF160887.1
H19	Muchuan 3#	Muchuan County, Sichuan Province	MG491269	FJ372919.1, KF160915.1, KF160887.1
H20	Xiangman 1#	Nanchong City, Sichuan Province	MG491264	KX394609.1, JQ780991.1, JQ731715.1
H21	Julu 1#	Julu County, Hebei Province	MG491265	KX394609.1, JQ780991.1, JQ731715.1

**Note:**

The selected reference sequences in GenBank have the highest identity with the sample sequence.

### Determination of active components

For active component analysis, the samples of each variety had three replications with 10 plants per replication collected from April to July 2015. In the current version of Ch.P (2015), the quality of LJF is primarily determined by the amount of chlorogenic acid and luteoloside content. Determined by the HPLC method, the content of chlorogenic acid should be higher than 1.5% and the content of luteoloside should be higher than 0.050% in dried products (Chinese Pharmacopoeia Commission, 2015). HPLC was conducted via a PerkinElmer system (Flexar PDA LC Detector, Totalchrom chromatographic work station) with an Agela C18 chromatographic column (250 × 4.6 mm, 5 μm).

### Data analysis

Forward and reverse sequences were spliced by DNAMAN software. The ITS sequences were aligned with Clustal X 1.83 (Thompson et al., 1997). The boundaries of the ITS region (ITS1-5.8S-ITS2) were determined by comparing homology with the aligned sequence previously published in the National Center of Biotechnology Information data base using Basic Local Alignment Search Tool (Altschul et al., 1990). The *L. japonica* referenced ITS sequences were EU240693.1, FJ774983.1, and FJ372911.1. The *L. macranthoides* referenced ITS sequences were FJ372922.1, FJ774984.1, and KF160887.1. All the ITS sequences of the 21 samples were submitted to GenBank and GenBank IDs were obtained (Table 1). *Weigela japonica* Thunb. (GenBank ID: EU240670.1)was used as the outgroup. Neighbor-Joining (NJ) trees were generated with the NJ method performed on a Kimura-2-parameter distance matrix with the MEGA 6.0 (Tamura et al., 2013) and carried out with 2,000 bootstrap replicates. Descriptive statistical analysis, analysis of variance (Duncan’s new multiple range test), Hierachical cluster analysis and *K*-cluster analysis (Jiang et al., 2014; Milev et al., 2015) of active components were performed using SPSS 19.0.

## Results

### Species identification and variation in ITS sequences

Basic Local Alignment Search Tool found that the ITS sequences of H10, H17, H18, and H19 were highly similar to *L. macranthoides*, based on identities ≥98% similar to the original plant of *Lonicerae* Flos (LF) in the Ch.P (Chinese Pharmacopoeia Commission, 2015). The other 17 varieties were highly similar to *L. japonica*, based on identities ≥99% similar. The reference sequences in GenBank are shown in Table 1.

The ITS regions including the 5.8S rDNA gene varied in their overall length from 610 bp (H17 and H19) to 618 bp (H10). The length of ITS1 sequences was 222–230 bp. The length of ITS2 sequence of all varieties was 225 bp. The 5.8S rDNA gene was 163 bp in length and the primary composition was identical in all varieties, reflecting the high conservativeness of 5.8S rDNA. Sequence comparisons (including the 5.8S rDNA gene) of the ITS regions of all varieties revealed a total of 618 nucleotides (excluding indels sites), of which 590 sites were constant, and 27 variable sites. Among variable sites, we determined 19 transitions and eight transversions, as well as 18 parsim-informative sites and nine singleton sites.

In *L. japonica*, ITS sequence analysis found that the ITS sequences of H1, H3, H6, H8, H9, H14, H15, and H20 were exactly identical. In contrast to the ITS sequences of these eight varieties, minor variations in the sequences of the remaining varieties were observed. For example, a deletion was discovered at position 6 of H21; a C-A single nucleotide polymorphism (SNP) at position 7 of H5; a C-G SNP at position 38 of H16, at position 121 of H2, H4, H7, H12, and H16; a G-A SNP at position 410 and 578 of H16 and H10; and a C-T SNP at position 417 of H2, H4, and H16, at position 459 of H13, and at position 568 of H11 and H13.

In *L. macranthoides*, the ITS sequences of H17, H18, and H19 were exactly identical. In contrast to the ITS sequences of H17, H18, and H19, in H10 we detected seven bp insertion (TCGAAAC) at the start of the ITS1 region; a C-A SNP at position 45 and 122, a T-C SNP at position 60, 182, 213, 214, and 568, and a A-G SNP at position 63, 69, 410, 453, and 601. H10 had A insertion at position 70 of the ITS1 region.

### Analysis of the ITS sequences

The intraspecific divergence values (Table 2) of *L. japonica* ranged from 0 to 0.012 (H13 and H16), among which the intraspecific divergence values of H1, H3, H5, H6, H8, H9, H14, H15, H20, and H21 varieties, as well as H2 and H4, H7 and H12, were 0. The intraspecific divergence values of *L. macranthoides* ranged from 0 (H17, H18, and H19) to 0.020 (H10). The interspecific divergence values of *L. japonica* and *L. macranthoides* ranged from 0.018 to 0.027, most of which were greater than the intra-specific divergence values.

**Table 2:** Genetic distances of all varieties based on Krima-2-Paramenter distance model.

	H1	H2	H3	H4	H5	H6	H7	H8	H9	H11	H12	H13	H14	H15	H16	H20	H21	H10	H17	H18	H19
H1																					
H2	0.003																				
H3	0.000	0.003																			
H4	0.003	0.000	0.003																		
H5	0.000	0.003	0.000	0.003																	
H6	0.000	0.003	0.000	0.003	0.000																
H7	0.002	0.002	0.002	0.002	0.002	0.002															
H8	0.000	0.003	0.000	0.003	0.000	0.000	0.002														
H9	0.000	0.003	0.000	0.003	0.000	0.000	0.002	0.000													
H11	0.002	0.005	0.002	0.005	0.002	0.002	0.003	0.002	0.002												
H12	0.002	0.002	0.002	0.002	0.002	0.002	0.000	0.002	0.002	0.003											
H13	0.003	0.007	0.003	0.007	0.003	0.003	0.005	0.003	0.003	0.002	0.005										
H14	0.000	0.003	0.000	0.003	0.000	0.000	0.002	0.000	0.000	0.002	0.002	0.003									
H15	0.000	0.003	0.000	0.003	0.000	0.000	0.002	0.000	0.000	0.002	0.002	0.003	0.000								
H16	0.008	0.005	0.008	0.005	0.008	0.008	0.007	0.008	0.008	0.010	0.007	0.012	0.008	0.008							
H20	0.000	0.003	0.000	0.003	0.000	0.000	0.002	0.000	0.000	0.002	0.002	0.003	0.000	0.000	0.008						
H21	0.000	0.003	0.000	0.003	0.000	0.000	0.002	0.000	0.000	0.002	0.002	0.003	0.000	0.000	0.008	0.000					
H10	0.022	0.022	0.022	0.022	0.022	0.022	0.023	0.022	0.022	0.020	0.023	0.018	0.022	0.022	0.020	0.022	0.022				
H17	0.025	0.025	0.025	0.025	0.025	0.025	0.027	0.025	0.025	0.027	0.027	0.025	0.025	0.025	0.027	0.025	0.025	0.020			
H18	0.025	0.025	0.025	0.025	0.025	0.025	0.027	0.025	0.025	0.027	0.027	0.025	0.025	0.025	0.027	0.025	0.025	0.020	0.000		
H19	0.025	0.025	0.025	0.025	0.025	0.025	0.027	0.025	0.025	0.027	0.027	0.025	0.025	0.025	0.027	0.025	0.025	0.020	0.000	0.000	

**Figure 1:**
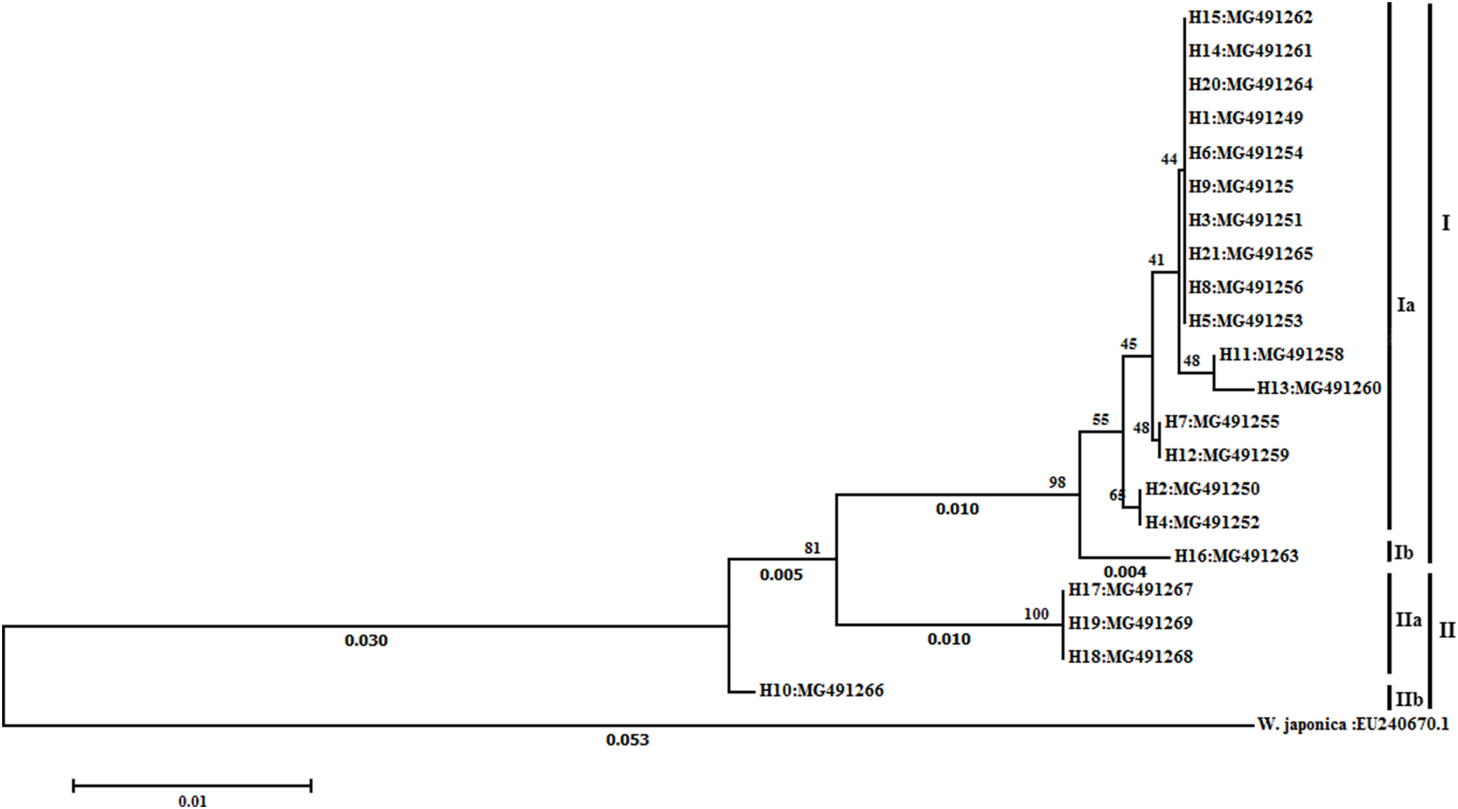
Phylogenetic tree resulted from Neighbor-Joining (NJ) analysis based on ITS sequences. Numerals below branches indicate branch length (hiden if shorter than 0.002). Numerals above branches indicate the percentage of times that the branch was recovered in 2,000 bootstrap replicates.

The NJ tree of all varieties are shown in Fig.1. Two main groups were identified by sequences with 81% bootstrap value. Group I consisted of 17 *L. japonica* varieties and group II consisted of four *L. macranthoides* varieties (H10, H17, H18, and H19). In group I, H16 was separated from the other 16 varieties with 98% bootstrap value. In group II, H10 was separated from H17, H18, and H19.

### Active component analysis

The tested flower bud samples were collected in the first year after introduction. Due to the difference of climate and soil characteristics between the varieties’ original provenance and the study area, H10 and H19 did not bloom, and H1, H2, H3, H4, H5, H6, H8, H9, H16, H17, H18 barely bloomed. Hence, the active component contents in the 13 varieties were not measured.

**Figure 2:**
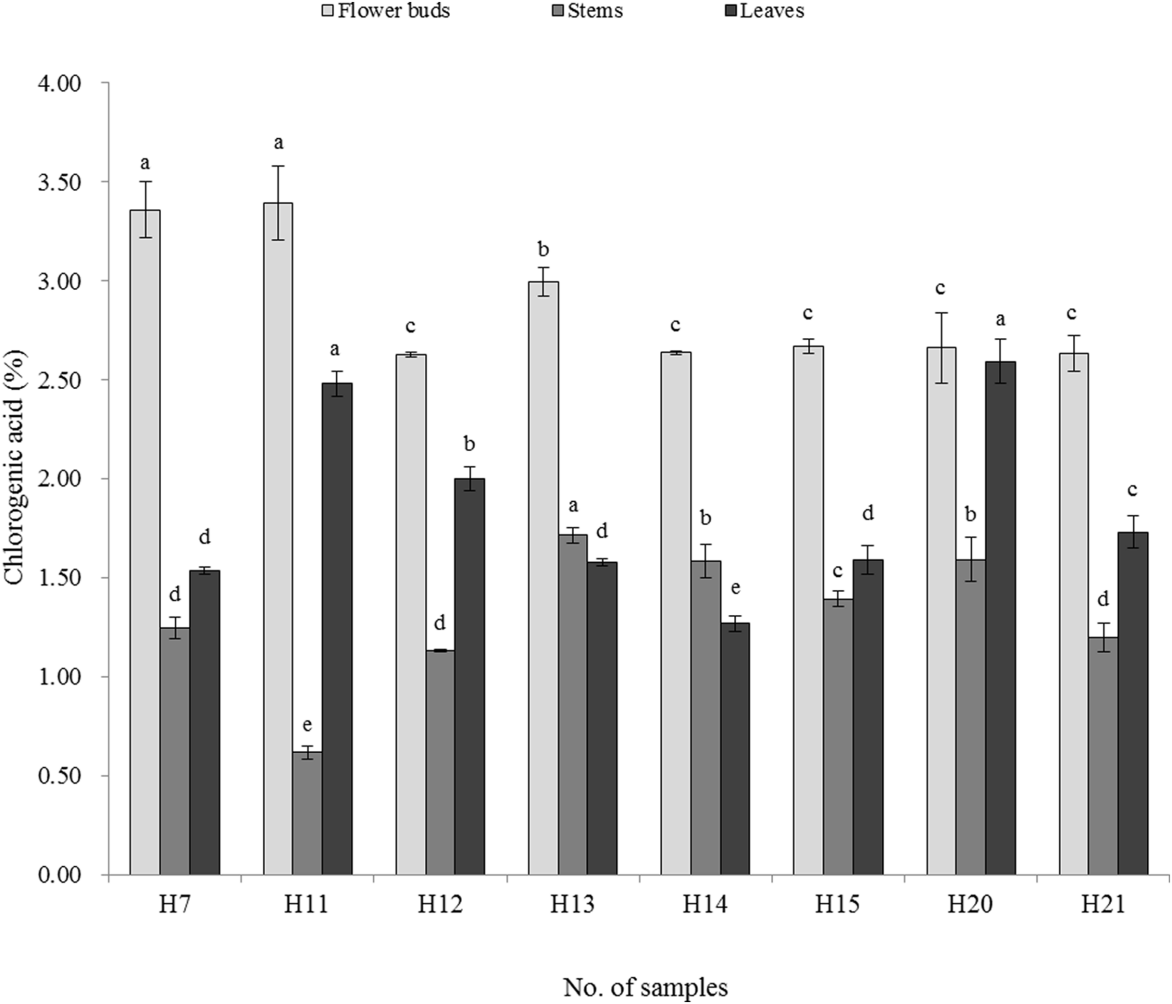
Content of chlorogenic acid of tested material (%). Different letters in the same sampling position indicate significant differences, determined by Duncan’s test with *p* < 0.05.

**Figure 3:**
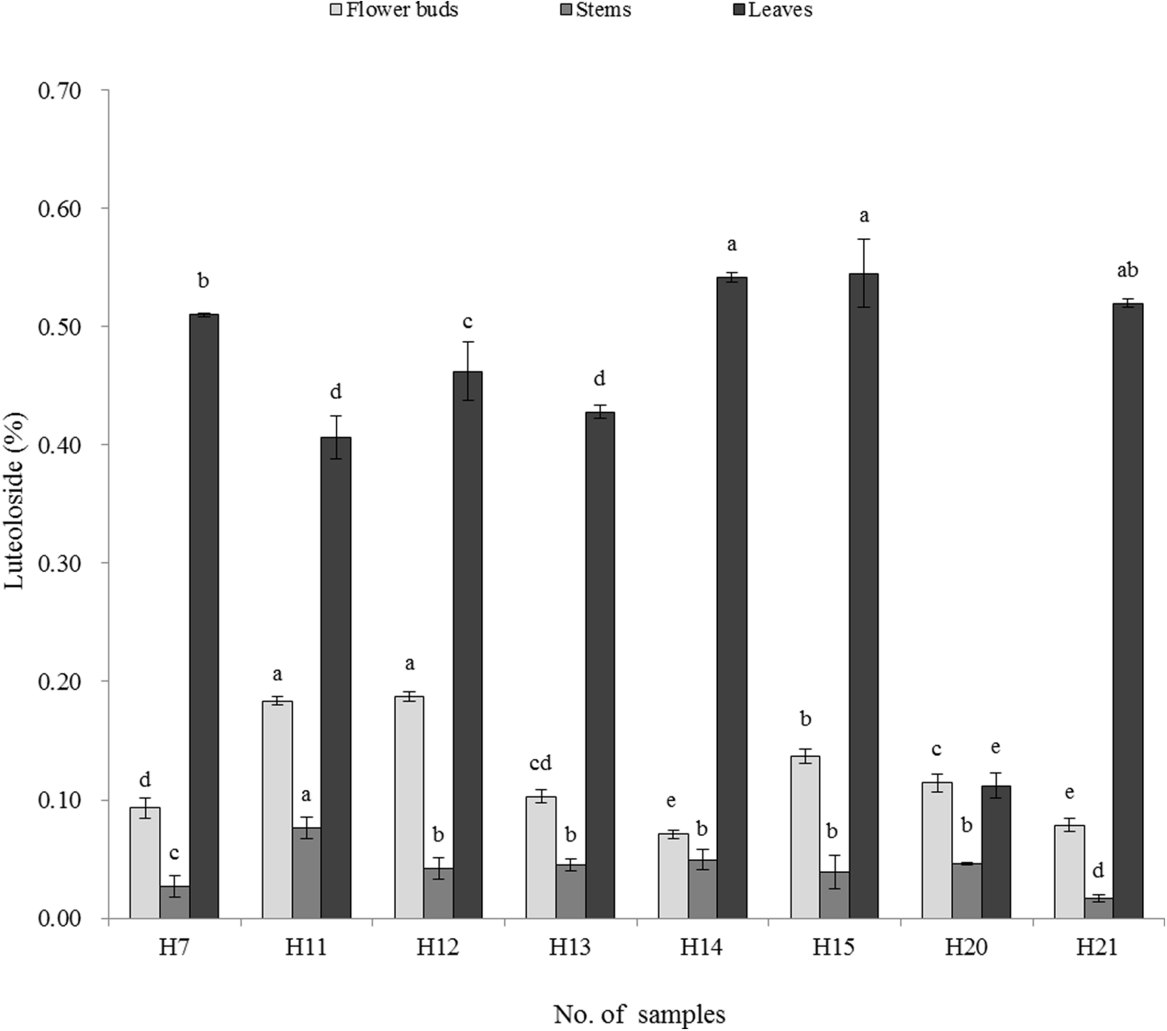
Content of luteoloside of tested material (%). Different letters in the same sampling position indicate significant differences, determined by Duncan’s test with *p* < 0.05.

The chlorogenic acid and luteoloside contents of LJF flower buds, stems and leaves were significantly different among the distinct LJF varieties (Figs. 2 and 3). In the flower buds, the highest chlorogenic acid content was detected in H11 (3.394%), while H12 had the lowest content (2.630%). The luteoloside contents ranged from 0.071% (H14) to 0.187% (H12). In the stems, the highest and lowest chlorogenic acid contents were detected in LJF cultivars of H13 (1.715%) and H11 (0.617%), respectively, while the highest and lowest luteoloside contents were detected in H11 (0.076%) and H21 (0.017%), respectively. In the leaves, the highest and lowest chlorogenic acid contents were detected in H20 (2.592%) and H14 (1.270%), respectively. The highest and lowest luteoloside contents were detected in H15 (0.545%) and H20 (0.112%), respectively.

**Figure 4:**
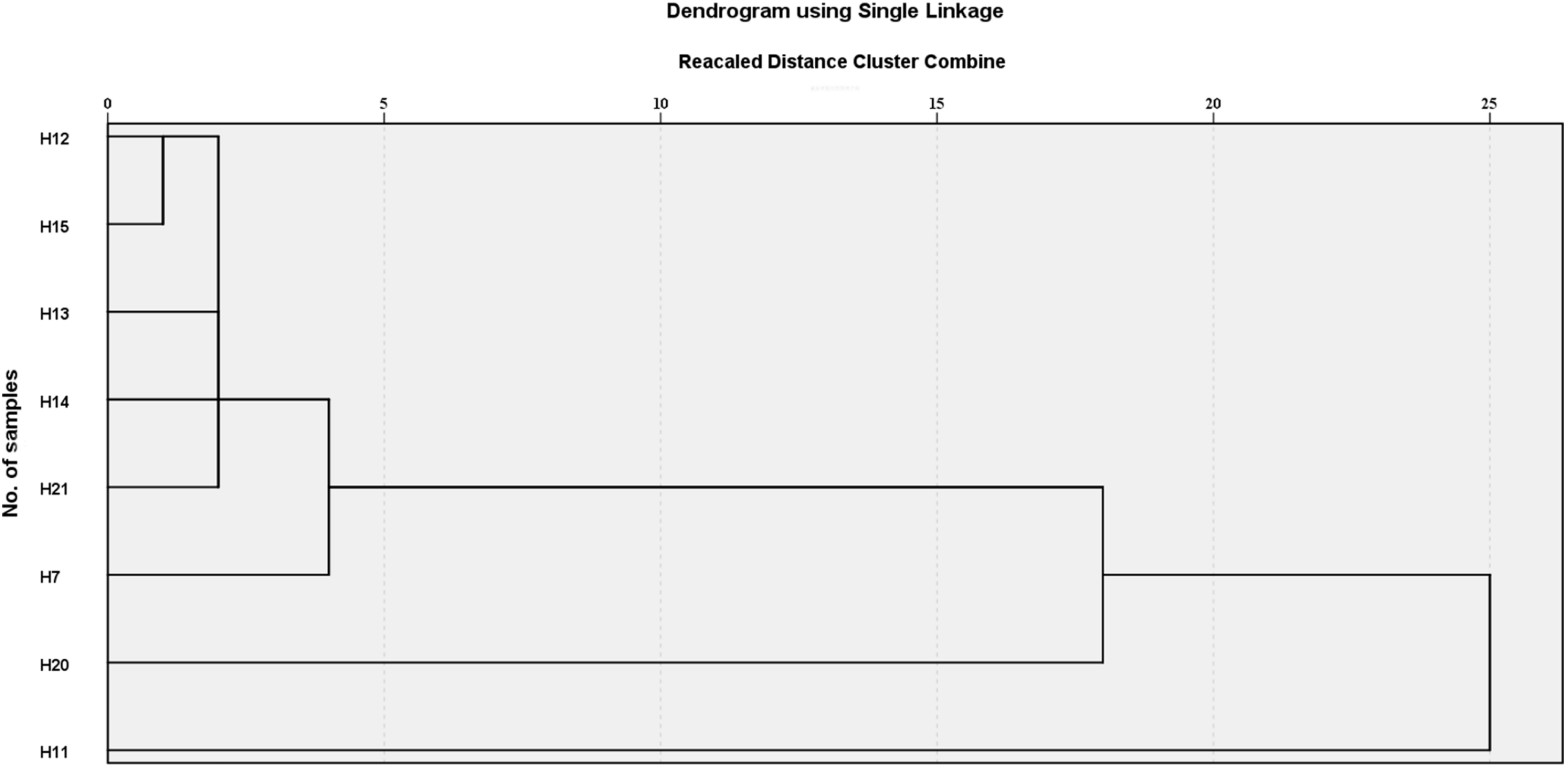
Hierachical cluster analysis of *L. japonicae*. Cluster method is nearest neighbor; interval measure is squared Euclidean distance; standardize: range 0–1.

Hierarchical cluster analysis was performed based on chlorogenic acid and luteoloside content in *L. japonica* flower buds, stems and leaves (Fig. 4). The results showed that the eight varieties could be divided into three categories when the squared Euclidean distance was 5. One category comprised H11, another comprised H20, and the last category held H7, H12, H13, H14, H15, and H21. The *K*-cluster analysis results were identical to the hierarchical clustering results (Table 3). One cluster contained H11, another contained H12 and H20, and one contained H7, H13, H14, H15, and H21. Except for the chlorogenic acid content in the stem and luteoloside content in the leaves that were slightly lower than in other varieties, the chlorogenic acid in the flower buds and leaves and luteoloside content in the flower buds and stems of H11 were significantly higher than in other varieties.

**Table 3:** Final cluster center of chlorogenic acid and luteoloside and cluster results of *L. japonicae*.

Clusters	Chlorogenic acid			Luteoloside			Results
	Flower buds	Stems	Leaves	Flower buds	Stems	Leaves	
1	2.860	1.427	1.541	0.097	0.035	0.509	H7, H13, H14, H15, H21
2	3.394	0.617	2.482	0.184	0.076	0.406	H11
3	2.646	1.362	2.295	0.151	0.044	0.287	H12, H20

## Discussion

About 200 species of *Lonicera* L. have been reported all over the world and nearly 50% of them (98 species, five subspecies and 18 variations) have been recorded in China (Hu et al., 2012). Owing to their similar morphological characteristics, it has been challenging to accurately identify the closely related species of *Lonicera* L. (Sun et al., 2011). Many wild *Lonicera* L. species are harvested and used as LJF. *L. japonica, L. hypoglauca, L. confusa*, and *L. dasystyla*, have been included in the Ch.P as the origin plant of LJF since 1977 (Ministry of Health of the People’s Republic of China, Commission of Pharmacopoeia, 1977, 1985, 1990, 1995; Chinese Pharmacopoeia Commission, 2000). In 2005, the flower buds of *L. macranthoides*, *L. hypoglauca*, and *L. confusa* were listed as LF in the Ch.P (Chinese Pharmacopoeia Commission, 2005) and *L. fulvotomentosa* was also included as one of the origin plants of LF in the 2010 and 2015 editions of Ch.P (Chinese Pharmacopoeia Commission, 2010, 2015). *L. japonica* is now recorded as the only origin plant of LJF, but LF is still regarded as LJF in some regions of China. From this study, H17, H18, and H19 are local LF cultivars that are used as LJF in Muchuan County, similar to H10 in Linyi City, implying that people should be more cautious when replacing or blending traditional Chinese medicines.

The accurate identification of a plant’s origin is the first step to assure quality of herbal medicines. DNA barcoding, a sequence-based method, is widely used for species identification. It has been found that using the psbA-trnH intergenic spacer as a DNA barcode is an efficient tool for the identification of *L. japonica* and its closely related species (Sun et al., 2011). Such barcoding allows distinguishing between botanical origins of LJF and LF, but it can also discriminate multi-origin species of LF (Sun et al., 2011). Seven candidate DNA barcodes (psbA-trnH, matK, rbcL, rpoC1, ycf5, ITS2, and ITS) from medicinal plant species were compared and it was found that the ITS2 of nrDNA represents the most suitable region to identify medicinal plants and their closely related species (Chen et al., 2010). Based on ITS sequences in this study, *L. japonica* was identified from *L. macranthoides*, and the genetic relationships between LJF and different *L. japonica* germplasm and *L. macranthoides* were clarified. Similar results have been reported in Peng et al. (2010), Hu et al. (2012) and Wang et al. (2014).

Internal transcribed spacer sequences of 16 LJF germplasm samples except H16 were clustered in group Ia, indicating that these samples may be of the same germplasm, but were named independently. Such a phenomenon is very common in LJF production in China. NJ analysis clearly separates H16 from the other LJF germplasm. H16 is a wild variety, while the other varieties are cultivars. The genetic distances between H16 and the other cultivated varieties (0.005–0.012) are greater than between cultivated varieties (0.000–0.003). It may be the long-term artificial cultivation and selection of LJF leading to such genetic variation. In addition, H2, H4, and H11 have the same phenotypic features with purple red buds, leaves and stems, while the other 14 varieties are the same with white corolla. Mature leaves of H4 have several small indentations. However, NJ analyses cluster the 16 varieties into one group. This reveals that the morphological classification of *L. japonica* differs from the molecular classification, because the gene controlling the color of the flower buds is not located in the ITS regions. Similar results have been reported by Maguire & Saenger (2000) and Du et al. (2015). Using leaf morphology and rDNA sequence data, Maguire & Saenger (2000) found that *Excoecaria dallachyana* Baillon was not closely related to mangrove species *E. agallocha* L. nor *E. ovalis* Endl, despite the superficial morphological similarities. However, Du et al. (2015) found that the *varietas* of *Salix matsudana* Koidz. had significant morphological differences, but only slight differences were detected at the molecular level. The varying results from distinct classification methods might be related to the morphological traits affected by growth conditions, as well as the selected feature. In *L. macranthoides*, H10 from Shangdong province was distinguished from HJ17, H18, and H19 from Sichuan province. Compared with H17, H18, and H19, the ITS sequence of H10 had multiple nucleotide site mutations. The result is similar to the analysis presented by Hu et al. (2012), in which *L. japonica* samples from four places were detached into two clades by other *Lonicera* L. species. The effects of physical isolation and differing environmental pressures (climate, soil, field management, and so on) may have led to significant variations in DNA sequences.

More than 140 compounds have been isolated and identified from LJF so far, and the main compositions are essential oils, organic acids, and flavones (Shang et al., 2011), in which the content of chlorogenic acid and luteoloside have been chosen as biomarkers to characterize the quality of LJF (Chinese Pharmacopoeia Commission, 2015). In this study, the content of chlorogenic acid and luteoloside varied in different *L. japonica* germplasm. The chlorogenic acid content of tested flower buds samples was higher than 1.5% and luteoloside content was higher than 0.05%. The chlorogenic acid and luteoloside contents were higher in red LJF flower (H11) buds than white LJF, similar to results presented in Yuan et al. (2012) and contrary to those presented in Wang et al. (2014). Two varieties (H10 and H19) introduced in the study area did not bloom, and 11 varieties (H1, H2, H3, H4, H5, H6, H8, H9, H16, H17, and H18) barely bloomed, showing the growth vigor differences in LJF and LF. All of the introduced LF varieties showed poor adaptability in the study area. In total, 16 varieties, except H16, were clustered into group Ia, which should be of LJF’s same germplasm with different sources. However, the growth vigor (H5 and H8 rarely bloomed) and the active components were significantly different, showing that the LJF varieties with different sources had different quality. Many studies have shown that the quality of LJF varied with habitat and varieties (Fan et al., 2015; Zhang, Feng & Wang, 2012; Rumalla et al., 2010; Qian et al., 2007; Chen et al., 2007), harvesting time and floral developmental stages (Jiang et al., 2014; Xiao & Li, 2011; Wang et al., 2009), and processing (Zhao et al., 2015). This indicates that, in order to reduce economic and time losses, one should be more cautious in large-scale growing of LJF. Studies have found that the chlorogenic acid content in the stems and leaves was lower than in the flower buds, and that the luteoloside content in the stems was lower than that in the flower buds. However, the luteoloside content in the leaves was apparently higher than that in the flower buds. Similar results have been reported by Wang et al. (2016). Therefore, the stems and leaves also have medicinal value and can used as an medicinal resource alternative to the flower bud. Notably, the benefits of *L. japonica* stems and leaves have been also reported by other studies (Ye et al., 2014; Xiong et al., 2013).

## Conclusions

The provided analysis of ITS sequences led to the successful identification of LF (*L. macranthoide*) and LJF from the mentioned varieties in the Hailuogou area. Based on cluster analysis using chlorogenic acid and luteoloside content in flower buds, stems and leaves, H11 was clustered separately with a high level of active components. H11 is a genuine *L. japonica* variety with a high medicinal value, and it should be introduced for large-scale planting in the Hailuogou area.

## Supplemental Information

10.7717/peerj.7636/supp-1 Supplemental Information 1 ITS sequences length (bp) of samples.

10.7717/peerj.7636/supp-2 Supplemental Information 2 Variable sites of ITS region in *Lonicera japonica* flos and *Lonicerae* flos. C is conserved sites, V is variable sites, Pi is parsim-informative sites, S is singleton sites.

10.7717/peerj.7636/supp-3 Supplemental Information 3 ITS sequences of tested samples. “.” indicate identical to H1, “-” indicate missing, A, T, G, or C indicate single nucleotide polymorphism (SNP)

10.7717/peerj.7636/supp-4 Supplemental Information 4 Content of chlorogenic acid and luteoloside of Sample (%). Different letters within the same column indicate significant differences, determined by D’s test with *p* < 0.05.

10.7717/peerj.7636/supp-5 Supplemental Information 5 Raw DNA sequence data in FAS format.

10.7717/peerj.7636/supp-6 Supplemental Information 6 Sequencing results.

10.7717/peerj.7636/supp-7 Supplemental Information 7 Variations of ITS.

## Additional Information and Declarations

### Data Availability

The following information was supplied regarding data availability: Data is available at NCBI: MG491249, MG491250, MG491251, MG491252, MG491253, MG491254, MG491255, MG491256, MG491257, MG491266, MG491258, MG491259, MG491260, MG491261, MG491262, MG491263, MG491267, MG491268, MG491269, MG491264, MG491265.
