# Supplementary material for: Identification and evaluation of Lonicera japonica flos introduced to the Hailuogou area based on ITS sequences and active compounds
Source: PeerJ. 2019 Sep 3;7:e7636. doi: 10.7717/peerj.7636 (PMC6730534; doi:10.7717/peerj.7636)
Supplement: Supplemental Information 1 [file peerj-07-7636-s001.docx]

| No. | ITS1 | 5.8S | ITS2 | ITS | No. | ITS1 | 5.8S | ITS2 | ITS |
| --- | --- | --- | --- | --- | --- | --- | --- | --- | --- |
| H1 | 229 | 163 | 225 | 617 | H12 | 229 | 163 | 225 | 617 |
| H2 | 229 | 163 | 225 | 617 | H13 | 229 | 163 | 225 | 617 |
| H3 | 229 | 163 | 225 | 617 | H14 | 229 | 163 | 225 | 617 |
| H4 | 229 | 163 | 225 | 617 | H15 | 229 | 163 | 225 | 617 |
| H5 | 229 | 163 | 225 | 617 | H16 | 229 | 163 | 225 | 617 |
| H6 | 229 | 163 | 225 | 617 | H17 | 222 | 163 | 225 | 610 |
| H7 | 229 | 163 | 225 | 617 | H18 | 229 | 163 | 225 | 617 |
| H8 | 229 | 163 | 225 | 617 | H19 | 222 | 163 | 225 | 610 |
| H9 | 229 | 163 | 225 | 617 | H20 | 229 | 163 | 225 | 617 |
| H10 | 230 | 163 | 225 | 618 | H21 | 228 | 163 | 225 | 616 |
| H11 | 229 | 163 | 225 | 617 |  |  |  |  |  |
