# Supplementary material for: Identification and evaluation of Lonicera japonica flos introduced to the Hailuogou area based on ITS sequences and active compounds
Source: PeerJ. 2019 Sep 3;7:e7636. doi: 10.7717/peerj.7636 (PMC6730534; doi:10.7717/peerj.7636)
Supplement: Supplemental Information 2 — C is conserved sites, V is variable sites, Pi is parsim-informative sites, S is singleton sites. [file peerj-07-7636-s002.docx]

|  |  | C | V | Pi | S | transition | transversion |
| --- | --- | --- | --- | --- | --- | --- | --- |
| All varieties | ITS1 | 213 | 16 | 7 | 9 | 8 | 8 |
|  | 5.8S | 163 | 0 | 0 | 0 | 0 | 0 |
|  | ITS2 | 214 | 11 | 11 | 0 | 11 | 0 |
|  | ITS | 590 | 27 | 18 | 9 | 19 | 8 |
| L. *japonica* | ITS1 | 226 | 3 | 1 | 2 | 1 | 2 |
|  | ITS2 | 220 | 5 | 2 | 3 | 5 | 0 |
| L.*macranthoides* | ITS1 | 217 | 12 | 0 | 8 | 5 | 3 |
|  | ITS2 | 221 | 4 | 0 | 4 | 3 | 1 |
