# Supplementary material for: Identification and evaluation of Lonicera japonica flos introduced to the Hailuogou area based on ITS sequences and active compounds
Source: PeerJ. 2019 Sep 3;7:e7636. doi: 10.7717/peerj.7636 (PMC6730534; doi:10.7717/peerj.7636)
Supplement: Supplemental Information 4 — Different letters within the same column indicate significant differences, determined by D’s test with p < 0.05. [file peerj-07-7636-s004.docx]

| NO. | Chlorogenic acid(%) | | |  | Luteoloside(%) | | |
| --- | --- | --- | --- | --- | --- | --- | --- |
|  | Flower buds | Stems | Leaves |  | Flower buds | Stems | Leaves |
| H7 | 3.360±0.142a | 1.247±0.055d | 1.538±0.019d |  | 0.093±0.008d | 0.027±0.009c | 0.510±0.001b |
| H11 | 3.394±0.189a | 0.617±0.033e | 2.482±0.064a |  | 0.184±0.004a | 0.076±0.009a | 0.406±0.018d |
| H12 | 2.630±0.012c | 1.133±0.008d | 1.998±0.061b |  | 0.187±0.005a | 0.042±0.009b | 0.462±0.025c |
| H13 | 2.996±0.073b | 1.715±0.039a | 1.579±0.017d |  | 0.103±0.005cd | 0.045±0.005b | 0.428±0.006d |
| H14 | 2.639±0.010c | 1.584±0.084b | 1.270±0.039e |  | 0.071±0.003e | 0.049±0.009b | 0.542±0.004a |
| H15 | 2.670±0.037c | 1.394±0.041c | 1.589±0.073d |  | 0.137±0.006b | 0.039±0.014b | 0.545±0.029a |
| H20 | 2.662±0.179c | 1.591±0.112b | 2.592±0.112a |  | 0.114±0.008c | 0.046±0.001b | 0.112±0.011e |
| H21 | 2.635±0.090c | 1.197±0.074d | 1.730±0.081c |  | 0.079±0.006e | 0.017±0.003d | 0.520±0.003ab |
