# Supplementary material for: Identification and evaluation of Lonicera japonica flos introduced to the Hailuogou area based on ITS sequences and active compounds
Source: PeerJ. 2019 Sep 3;7:e7636. doi: 10.7717/peerj.7636 (PMC6730534; doi:10.7717/peerj.7636)
Supplement: Supplemental Information 7 [file peerj-07-7636-s007.docx]

| Position | Polymorphism type | Reference | H1 | H2 | H3 | H4 | H5 | H6 | H7 | H8 | H9 | H11 | H12 | H13 | H14 | H15 | H16 | H21 |
| --- | --- | --- | --- | --- | --- | --- | --- | --- | --- | --- | --- | --- | --- | --- | --- | --- | --- | --- |
| 6 | Deletion | A |  |  |  |  |  |  |  |  |  |  |  |  |  |  |  | Deletion |
| 7 | SNP | C |  |  |  |  | A |  |  |  |  |  |  |  |  |  |  |  |
| 38 | SNP | C |  |  |  |  |  |  |  |  |  |  |  |  |  |  | G |  |
| 121 | SNP | C |  | G |  | G |  |  | G |  |  |  | G |  |  |  | G |  |
| 410 | SNP | G |  |  |  |  |  |  |  |  |  |  |  |  |  |  | A |  |
| 417 | SNP | C |  | T |  | T |  |  |  |  |  |  |  |  |  |  | T |  |
| 459 | SNP | C |  |  |  |  |  |  |  |  |  |  |  | T |  |  |  |  |
| 568 | SNP | C |  |  |  |  |  |  |  |  |  | T |  | T |  |  |  |  |
| 578 | SNP | G |  |  |  |  |  |  |  |  |  |  |  |  |  |  | A |  |

Table 1 Variations in the *L. japonica*

Table 2 Variations in the *L. macranthoides*

| Position | Polymorphismtype | Reference | H10 | H18 | H19 | H20 |
| --- | --- | --- | --- | --- | --- | --- |
| 1-7 | SNP | TCGAAAC | Deletion |  |  |  |
| 48 | SNP | A | C |  |  |  |
| 60 | SNP | C | T |  |  |  |
| 63 | SNP | G | A |  |  |  |
| 69 | SNP | G | A |  |  |  |
| 70 | Insertion |  | A |  |  |  |
| 122 | SNP | A | C |  |  |  |
| 182 | SNP | C | T |  |  |  |
| 213 | SNP | C | T |  |  |  |
| 214 | SNP | C | T |  |  |  |
| 410 | SNP | G | A |  |  |  |
| 453 | SNP | G | A |  |  |  |
| 568 | SNP | C | T |  |  |  |
| 601 | SNP | G | A |  |  |  |
